# Deep-Learning-Based Cerebral Artery Semantic Segmentation in Neurosurgical Operating Microscope Vision Using Indocyanine Green Fluorescence Videoangiography

**DOI:** 10.3389/fnbot.2021.735177

**Published:** 2022-01-12

**Authors:** Min-seok Kim, Joon Hyuk Cha, Seonhwa Lee, Lihong Han, Wonhyoung Park, Jae Sung Ahn, Seong-Cheol Park

**Affiliations:** ^1^Clinical Research Team, Deepnoid, Seoul, South Korea; ^2^Department of Internal Medicine, Inha University Hospital, Incheon, South Korea; ^3^Department of Bio-convergence Engineering, Korea University, Seoul, South Korea; ^4^Department of Computer Science and Engineering, Soongsil University, Seoul, South Korea; ^5^Department of Neurosurgery, Asan Medical Center, University of Ulsan College of Medicine, Seoul, South Korea; ^6^Department of Neurosurgery, Gangneung Asan Hospital, University of Ulsan College of Medicine, Gangneung, South Korea; ^7^Department of Neurosurgery, Seoul Metropolitan Government—Seoul National University Boramae Medical Center, Seoul, South Korea; ^8^Department of Neurosurgery, Hallym Hospital, Incheon, South Korea

**Keywords:** semantic segmentation, neural network, blood vessel, indocyanine green, neurosurgical field, computer vision, deep learning, cerebral artery

## Abstract

There have been few anatomical structure segmentation studies using deep learning. Numbers of training and ground truth images applied were small and the accuracies of which were low or inconsistent. For a surgical video anatomy analysis, various obstacles, including a variable fast-changing view, large deformations, occlusions, low illumination, and inadequate focus occur. In addition, it is difficult and costly to obtain a large and accurate dataset on operational video anatomical structures, including arteries. In this study, we investigated cerebral artery segmentation using an automatic ground-truth generation method. Indocyanine green (ICG) fluorescence intraoperative cerebral videoangiography was used to create a ground-truth dataset mainly for cerebral arteries and partly for cerebral blood vessels, including veins. Four different neural network models were trained using the dataset and compared. Before augmentation, 35,975 training images and 11,266 validation images were used. After augmentation, 260,499 training and 90,129 validation images were used. A Dice score of 79% for cerebral artery segmentation was achieved using the DeepLabv3+ model trained using an automatically generated dataset. Strict validation in different patient groups was conducted. Arteries were also discerned from the veins using the ICG videoangiography phase. We achieved fair accuracy, which demonstrated the appropriateness of the methodology. This study proved the feasibility of operating field view of the cerebral artery segmentation using deep learning, and the effectiveness of the automatic blood vessel ground truth generation method using ICG fluorescence videoangiography. Using this method, computer vision can discern blood vessels and arteries from veins in a neurosurgical microscope field of view. Thus, this technique is essential for neurosurgical field vessel anatomy-based navigation. In addition, surgical assistance, safety, and autonomous surgery neurorobotics that can detect or manipulate cerebral vessels would require computer vision to identify blood vessels and arteries.

## Introduction

### Research Background and Key Points

#### Previous Related Studies

- Artery segmentation using neurosurgical operating microscope video is mostly unexplored, partially owing to the highly variant morphology, various obstacles to surgical video segmentation, and difficulty in achieving a sufficient and accurate dataset.
- In a few studies, instrument segmentation in the surgical field has shown a higher accuracy than anatomical structure segmentations.

#### Value of This Study

- Indocyanine green (ICG) fluorescence intraoperative cerebral videoangiography can be used for automatic data creation for deep-learning-based artery semantic segmentation, unlike most previous surgical video segmentation studies dependent on manual markings. The arterial ground truth can also be distinguished from veins using the ICG videoangiography phase.
- The performances of the models trained using our method are convincing.

#### Potential Applications

- Indocyanine green (ICG) fluorescence intraoperative cerebral videoangiography has the potential to be developed into a valuable method for a more easily achievable quality of datasets.
- Neurosurgical operating microscope video cerebral artery segmentation is important for future vision-based navigation surgical assistance and autonomous surgery using artificial intelligence.

### Backgrounds of Cerebral Blood Vessel Segmentation

Deep learning algorithms have been successfully applied to medical images, such as in an MRI analysis (Doke et al., 2020; Wang et al., 2020). In previous surgical video analysis studies using deep learning and pixel-wise instrument segmentations were possible, achieving a fair level of accuracy with a neurosurgical microscope (Kalavakonda, 2019) or laparoscopic datasets (Kamrul Hasan and Linte, 2019). In recent studies, for surgical instrument segmentation, the mean Dice score was ~0.769–0.9 (Kalavakonda, 2019; Kamrul Hasan and Linte, 2019).

However, studies on anatomical structure segmentation using neurosurgical microscope operational video are rare (Jiang et al., 2021). This is probably because anatomical features are more difficult to accurately segmentize through machine learning. A recent study on anatomy segmentation using a neurosurgical operating microscope focused on manual ground truth marking methods, and results of the analysis have yet to be reported (Pangal et al., 2021). In a recent study, U-net-based vessel segmentation was reported not for visible light color neurosurgical video but for grayscale infrared ICG videoangiography vessels (Jiang et al., 2021). In addition, the main focus was blood flow analysis, and only 150 cortical vessel images were used for training (Jiang et al., 2021).

Instead, some related studies have focused on different surgical views or tissues, such as laparoscopic (Bamba et al., 2021), fetoscopic views (Bano et al., 2021), laryngeal tissue (Laves et al., 2019), and uterus and ovaries (Madad Zadeh et al., 2020). The overall mean intersection over union (IoU) reported for anatomical structures has often been as low as 0.56 (Bamba et al., 2021) or within the range of 0.58–0.69 (Bano et al., 2021).

Numerous difficulties with surgical video instruments and anatomical feature segmentation have been described in the literature, including anatomical variability, dynamical changes in three-dimensional viewpoints, few differences in texture, limited resolutions, occlusions, high tissue deformations, shadows, and reflections (Kalavakonda, 2019; Bamba et al., 2021; Bano et al., 2021). As a result, datasets of surgical video features are likely much more difficult to analyze than the previously investigated datasets with more constant vessels, for example, retinal blood vessels (Khanal and Estrada, 2020).

Almost all previous operative anatomy segmentation studies depend on manual ground truth markings (Kalavakonda, 2019; Madad Zadeh et al., 2020; Bamba et al., 2021; Bano et al., 2021; Pangal et al., 2021). However, achieving the anatomical structure ground truth through manual markings is time-consuming, expensive, and potentially inaccurate. This is partially because the surgical field of view annotations are sometimes difficult to achieve and are often created manually by a few expert surgeons (Madad Zadeh et al., 2020). Even in recent studies, researchers have continued to make efforts to create a consistent manual annotation of neurosurgical operation videos, and segmentation has yet to be achieved (Pangal et al., 2021). Some helper software such as “Supervisely” (Madad Zadeh et al., 2020; Bano et al., 2021; Pangal et al., 2021) has been used in the annotating process. However, this method requires manual processing. Furthermore, costly and inaccurate manual annotation results prevented obtaining high-quality data.

To overcome this problem, we first used ICG fluorescence intraoperative cerebral videoangiography to efficiently and accurately create a ground-truth dataset for blood vessels. We generated ground-truth data from infrared videos captured through operating microscope views. This is a novel and more autonomous method of dataset annotation for operating microscope-view cerebral arteries segmentation. Thus, we can achieve a more abundant and accurate dataset.

In this study, we focused on cerebral arteries in the neurosurgical field. To clearly discern the arteries, the artery ground truth must be distinguished from the veins. We were able to distinguish the artery ground truth from veins using the ICG videoangiography phase.

In addition, recent research into advanced neural network architectures used in image semantic segmentation may improve the semantic segmentation of anatomical structures (Wang et al., 2020). In previous studies, earlier architectures, such as U-Net (Kamrul Hasan and Linte, 2019; Sadda et al., 2019; Jiang et al., 2021), were adopted. However, recent architectures, such as DeepLabv3+ (Chen et al., 2018) or convolutional neural networks with modified or deeper network architectures, may achieve a better performance (Wang et al., 2020). We reported the evaluations and comparative performances of segmentation architectures on blood vessels captured through neurosurgical microscope operation videos.

The algorithm hyperparameters and optimization methods are also important (Doke et al., 2020). The performance of the algorithm according to the hyperparameters was also investigated in this study. The main contributions of this study are proof of concept regarding the effectiveness of an ICG videoangiography-based ground truth generation method and the feasibility of achieving cerebral artery segmentation through deep learning.

## Materials and Methods

### Recording Videos

Indocyanine green (ICG) intraoperative cerebral videoangiography has been used (Bruneau et al., 2013; Norat et al., 2019). In addition, ICG infrared videoangiography is a frequently used technique to assess vascular patency and occlusions during cerebrovascular surgeries (Norat et al., 2019).

**Figure 1:**
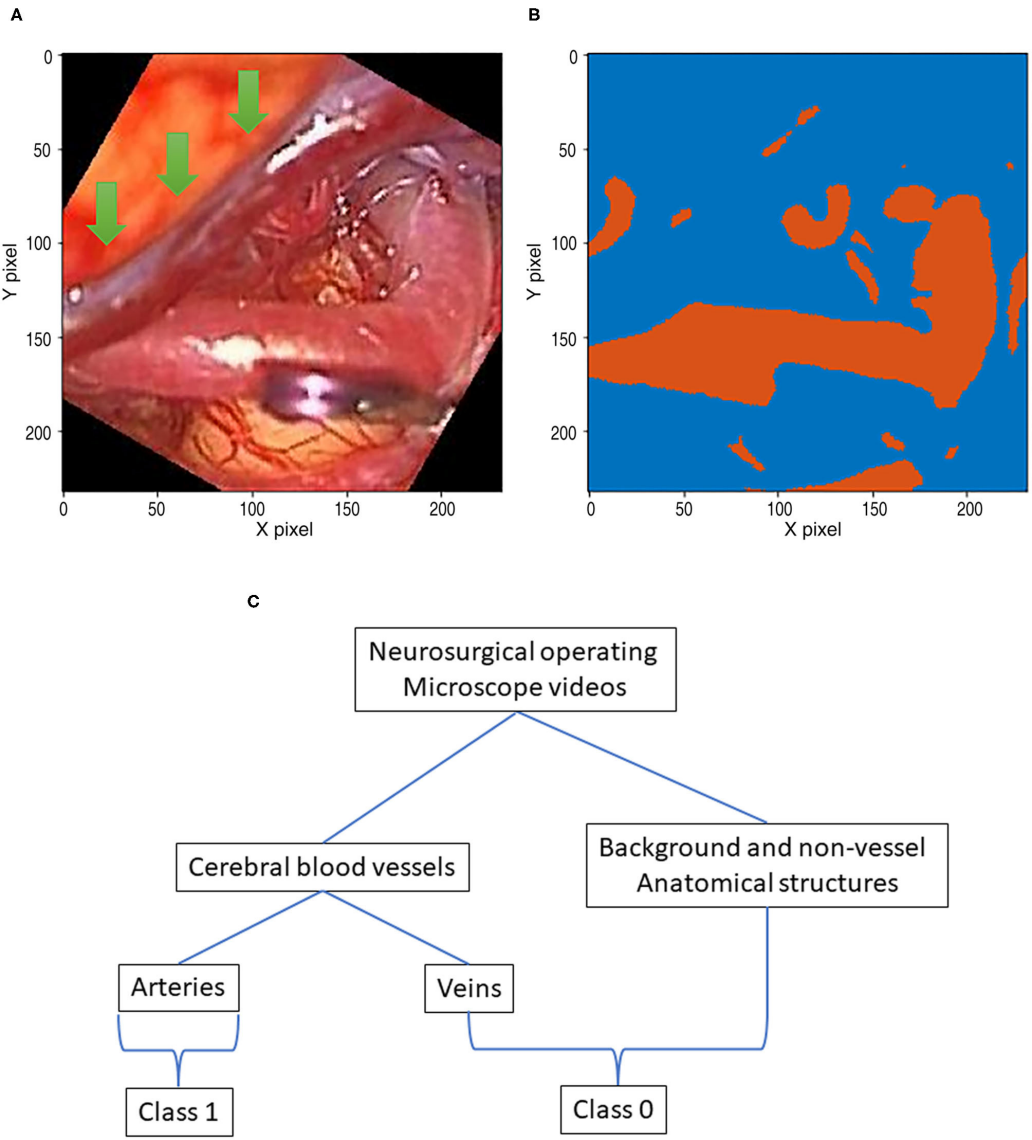
A data pair of full color visible light image data and artery ground truth map data generated from neurosurgical operating microscope ICG fluorescence videoangiography (IR800). Large veins with darker color are excluded (green arrows). **(A)** Visible light image. **(B)** Ground truth map from ICG. **(C)** Deep learning semantic segmentation class allocation according to anatomical structure and object types.

Training the semantic segmentation model requires image pairs that are used for the input of the model and ground truth images, which generally contained label-indexed pixels (Figure 1). Because some neurosurgical operative microscopes can simultaneously record both visible ray color images and infrared ICG videoangiography images, which can be used to label vessels, we used ICG intraoperative cerebral videoangiography for the autonomous generation of ground truth images with minimal manual setting adjustments.

Videos were recorded using a neurosurgical operating microscope. Videos with different resolutions, i.e., high definition (HD) 1920 × 1080 videos from an OPMI Pentero 900 (ZEISS, Oberkochen) and standard definition (SD) 720 × 480 videos from OPMI Pentero 800 (ZEISS, Oberkochen), were obtained. Both visible light and infrared videos were shot at 29.97 frames per second. Dual video recording, under both visible light and 800 nm wavelength infrared light, is a function of the operating microscope manufacturer, ZEISS, and is referred to as “IR800.” Two types of visible-light and infrared cameras were embedded within the OPMI Pentero microscope. Full-color visible-light videos were used as input images, and infrared (800 nm) ICG videoangiography videos (IR800) (ZEISS, Oberkochen) were used to create ground truth images. Commonly, only visible light videos are recorded. When the IR800 function is turned on through a user-selected button on the microscope, both types of videos are recorded. These videos are stored as separate files within the computer storage of the operating microscope. When the IR800 function is turned on, the microscope monitor shows infrared images, and the viewfinder still shows visible-light images. These videos can be copied through an operating microscope USB port with graphical user interface menu buttons. These visible light and infrared videos are mostly matched in their timing and viewpoints. However, these matches are not perfect, and some preprocessing is required. The dataset was collected from a total of 99 patient videos based considering video quality.

### Brightness Variability and Thresholding of ICG Fluorescence Infrared Videos

First, thresholding was conducted as a pre-processing step for ICG infrared fluorescence videoangiography grayscale videos. Because such raw video data are in grayscale, they need to be thresholded to create a ground-truth image with a class of zero for the background or a class of 1 for the artery pixels (Figure 1C) for semantic segmentation training using deep learning.

The brightness of the ICG fluorescence is influenced by multiple factors. An ICG dye was injected intravenously immediately before the videoangiography. The blood volume, dye dilution, vessel atherosclerosis, wall thickness, and blood flow speed influence the videoangiography (Norat et al., 2019).

Indocyanine green (ICG) videoangiography is based on “fluorescence” and not “luminescence,” and ICG does not emit photons by itself. Instead, a light source from the operating microscope is required to emit fluorescence (Norat et al., 2019). Thus, the brightness of fluorescence is also influenced by the amount of illumination from the operating microscope. The amount of illumination is also influenced by the surgical field of depth, magnification, and light source aperture of the operating microscope, and various soft tissue and instrument occlusions, including brain cortices, retractors, other instruments, and the surgeon's hand, all resulting in shadows. These factors also influence the image quality and potentially the accuracy of the analysis.

Finally, the brightness of ICG fluorescence videoangiography showed moderate variability among the patients. Before thresholding, we used the “adeqhisteq” function of MATLAB 2019b (MathWorks, Inc., Natick, Massachusetts, United States) to improve the image contrast for better thresholding. This function uses contrast-limited adaptive histogram equalization (Pizer, 1990). For thresholding, the “imbinarize” MATLAB function was used. When there is no option, “imbinarize” uses Otsu's method for thresholding (Otsu, 1979). When the threshold option is present, the designated threshold is used in the “imbinarize” function. In an early study, we used Otsu's thresholding (Otsu, 1979). However, for a minority of patients, this thresholding method is inadequate for threshold vessels. The fixed thresholds are then used. A threshold of ~0.55, i.e., between 0.5 and 0.6, was adequate for 98% of the patients. A value of 0.55 was used for 58% of the patients, while values of 0.5 and 0.6 were used for 20% of the patients each. Finally, 0.7 was used for one patient.

### Temporal Matching Between Visible Light and ICG Fluorescence Infrared Videos

Temporal offsets between the visible light view frame and the videoangiography view frame were adjusted to match. The video record timings of visible light and ICG infrared videos are extremely close. However, they often differ by a few frames. The start of color visible light videos is slower than ICG infrared fluorescence videos by ~3 or 10 frames for SD videos and 8 frames for HD videos. The frame rates for both types of videos were 29.97 fps.

### Image Cropping Area Selection Considering Focus and Quality

Typically, the neurosurgical operation field is narrow and as deep as several to more than 10 cm from the cerebral cortex. Thus, only a small portion of the entire video field is correctly focused, adequately illuminated, and has high-quality cerebral vessel images. Thus, only cropped image patches were used for deep learning training. Images with various types of inappropriate conditions, including a lack of light, difficulty in focusing, limited vision, and inappropriate synchronization with infrared fluorescence ICG videoangiography videos, were excluded (Kalavakonda, 2019; Bano et al., 2020, 2021; Bamba et al., 2021). Owing to the limited image qualities of the variable intraoperative surgical field of view, the selection of appropriate scenes is required in our study. The acquired images can be classified into various characteristic groups according to the video quality. Although all characteristics were used Supplementary Table 1 for training and evaluation, images of poor quality were excluded. In the pre-selection step, the appropriate frames were chosen from the videos.

Finally, some types of cerebral vessels were inadequate for the analysis. Cortical capillaries or small perforators have substantially different morphologies compared to deeply located medium and large-sized arteries. These types need to be trained in separate categories and excluded from this study. Thus, we only selected image patches that mainly contained medium and large-sized arteries. The focus was placed on large and mid-sized, non-atherosclerotic blood vessels, for example, arteries including the middle cerebral artery M1, M2 and M3 branches, and anterior cerebral artery A1, A2, and A3 branches and arterioles, which can be conspicuously visualized through ICG fluorescence videoangiography. Atherosclerotic yellowish internal carotid arteries (ICA) were excluded. This was because the ICG fluorescence came from circulating blood, often, ICG florescence only partly pass the thick atherosclerotic ICA walls or were unable to do so at all. The image resolution of a patch is smaller than the whole video field, potentially limiting the accuracy of the analysis.

### Spatial Matching Between Visible Light and ICG Fluorescence of Infrared Videos

When SD videos are used, the ICG infrared fluorescence camera also has an SD resolution, and no magnification is required. However, images of infrared fluorescence ICG videoangiography and surgical images of full-color visible light have different resolutions when HD videos are used. The horizontal and vertical magnification ratios differ for the two types of videos. Infrared videos were magnified 2.28 times in the horizontal axis and 2.43 times in the vertical axis. These ratios were then fixed to the microscope model.

Visible light video cameras and infrared video cameras are two different types of cameras embedded within a microscope. Thus, the two videos were not exactly matched. Instead, the relationship between the two videos created a stereovision. Because of the stereovision relationship, spatial offsets and distortions existed in both videos. These spatial offsets are not constant within the entire video field and may differ according to the depth of field and viewpoint of the operating microscopes. Occasionally, the neurosurgeon might move the operating microscope during the recording of the ICG infrared fluorescence videoangiography, resulting in a change in camera viewpoints. Theoretically, the stereovision relationship can be calculated. However, we used image patches, and not the entire video field, and we only needed the matching of a small number containing a high-quality vessel image. In small areas, the errors from stereovision distortions were extremely small. Therefore, we adopted a simpler method, i.e., a 2D co-registration, for matching the image patches between visible light color images and infrared fluorescence images. In particular, for image registration, the MATLAB image processing toolbox 2D co-registration function “imregister” optimizer was used, and a similarity transformation was chosen as a co-registration algorithm.

### Segmentation Class Allocation According to ICG Fluorescence Videoangiography Phase Selection and Datasets

Indocyanine green (ICG) flows from the arteries to the veins. Thus, in the early phases of ICG videoangiography, only the arteries were visualized. We refer to this timing as the arterial phase in this study. Both arteries and veins were visualized during the delayed phase. Only arterial-phase ICG videoangiography was mainly used; thus, veins with slightly different morphologies were not targeted for the segmentation in this study (Figure 1C). Therefore, our segmentation target excluded almost all non-vessel anatomical structures and veins (Figure 1C). However, for comparison, in a separate analysis, a small portion of the delayed phase, including both arteries and veins, was used.

A dataset containing only the arterial phase, along with 92 patients including 84 patients from the training dataset of 34,348 images, and 8 patients from the test dataset with 7,933 images without augmentation, was analyzed.

For comparison, in the dataset including a delayed phase, the 99 patients included 84 patients with an artery phase and 4 patients with a delayed phase in the training dataset, which has 35,975 images without augmentation. Delayed phases were used in 3 of the 11 test group patients with 11,266 images without augmentation.

When an appropriate image frame is found, ~60 frames are selected for the front and back.

### Data Augmentation

**Figure 2:**
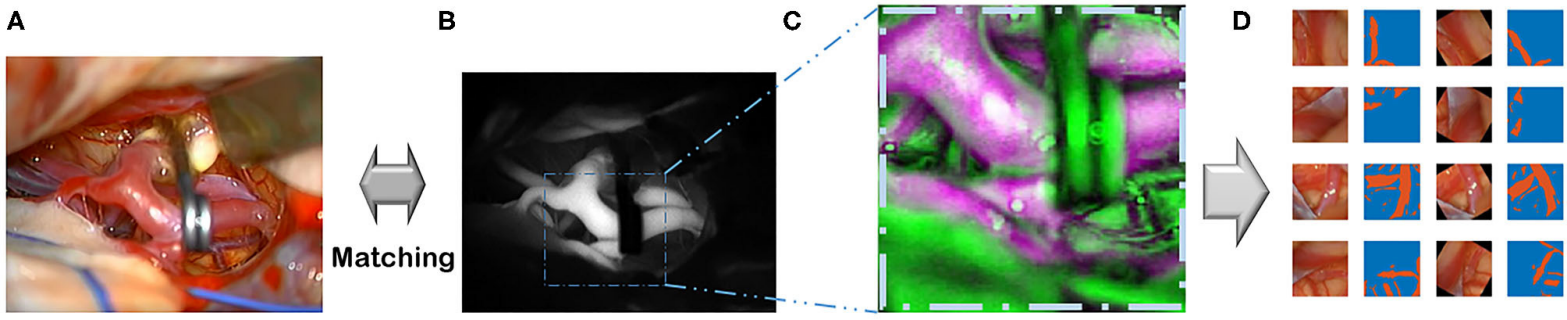
This flowchart demonstrates the pipeline, which includes recording, pre-processing, and augmentation. **(A)** Recording visible ray color video. **(B)** ICG infrared fluorescence videos. **(C)** Image co-registration of color images. **(D)** Augmentations are done.

Images from the diverse random conditioned frames were cropped within a selected field of video frames using MATLAB during the training image generation (Figure 2). In addition, a rotation augmentation was applied to the image pair using a 45° delta.

In the 92-patient group with all artery phases, for the training dataset containing 84 patients, 34,348 images were augmented into 251,086 images. For the test dataset containing 8 patients, 7,933 images were augmented into 60,849 images. In the 99-patient group, with 92 patients had an artery phase and 7 patients had a delayed phase. For the training dataset containing 88 patients with 4 patients having a delayed phase, 35,975 images were augmented into 260,499 images. For the test dataset containing 11 patients with 3 patients having a delayed phase, 11,593 images were augmented into 90,129 images. For appropriate deep learning architecture inputs, all pairs were resized to a pixel resolution of 512 × 512.

### Network Architectures

A fully convolutional network (FCN) (Shelhamer et al., 2017) is a base network created by changing the last linear layer of Visual Geometry Group (VGG) network (Simonyan and Zisserman, 2014) into a 1 × 1 convolution layer and by adding upscaling to predict each pixel. U-Net is a type of network that uses skip connections and concepts related to residuals (Ronneberger et al., 2015). DeepLabv3 (Chen et al., 2017) and v3+ (Chen et al., 2018) are architectures that adopt an atrous convolution to handle more sparsely dispersed features. These three architectures, FCN, DeepLabv3 and DeepLabv3+, were trained on the same backbone network, ResNet-101, unlike U-Net. The TorchVision-pretrained ResNet-101 model was chosen, which was trained with data having mean values of 0.485, 0.456, and 0.406 and standard deviations of 0.229, 0.224, and 0.225. For an FCN, which was originally based on VGG, the last feature of ResNet-101 was used, which has 2,048 channels, and a simple version of the FCN head was constructed (Table 1). All architectures produced binary channel maps of the same size as the input image. The original DeepLabv3+ employed various backbone networks (Chen et al., 2018), and the ResNet-101 backbone was used in this study.

**Table 1:** Deeplab V3+ header architecture based on ResNet-101.

**Layer**	**Output size**	**Filter size**	**Stride**	**Padding**	**Dropout**
ResNet-101 low level feature	256 × 128 × 128	–	–	–	–
Convolution 1	48 × 128 × 128	1 × 1	1	1	0.1
ResNet-101 out feature	2,048 × 64 × 64	–	–	–	–
Atrous spatial pyramidal pooling	1,280 × 64 × 64	–	–	–	–
Project convolution	256 × 64 × 64	1 × 1	1	–	0.1
Up sampling (interpolate)	256 × 128 × 128	–	–	–	–
Concatenation	304 × 128 × 128	–	–	–	–
Convolution	256 × 128 × 128	3 × 3	1	1	–
Convolution	2 × 128 × 128	1 × 1	1	–	–
Up sampling (interpolate)	2 × 512 × 512	–	–	–	–

### Training Details

A pretrained ResNet-101 was used, which was published using the TorchVision models. The 2D cross entropy was calculated, and the stochastic gradient descent has updated this loss metric based on the learning rate with a weight decay. The models used for comparison were selected through the intersection over union (IoU) on the validation dataset. All models were implemented using PyTorch. A single graphics processing unit (GPU) (NVIDIA GeForce GTX 1080 Ti) with 11 GB of VRAM was used for each training. Seven GPUs were used to conduct the training and validation under various training hyperparameters and analysis settings based on previous studies (Smith, 2018; Doke et al., 2020). The models were trained using hyperparameters including a 10^−8^ learning rate, 5 × 10^−5^ weight decay, and 0.99 momentum. All models and various other hyperparameter settings were trained for at least 50,000 iterations and up to 100,000 iterations. All architectures are the same as in the original paper, excluding the FCN, as described in Table 1. The output stride for DeepLabv3+ was fixed at 8, considerably better results than those with larger strides found in the literature (Shelhamer et al., 2017; Chen et al., 2018).

### Evaluation Protocol

**Figure 3:**
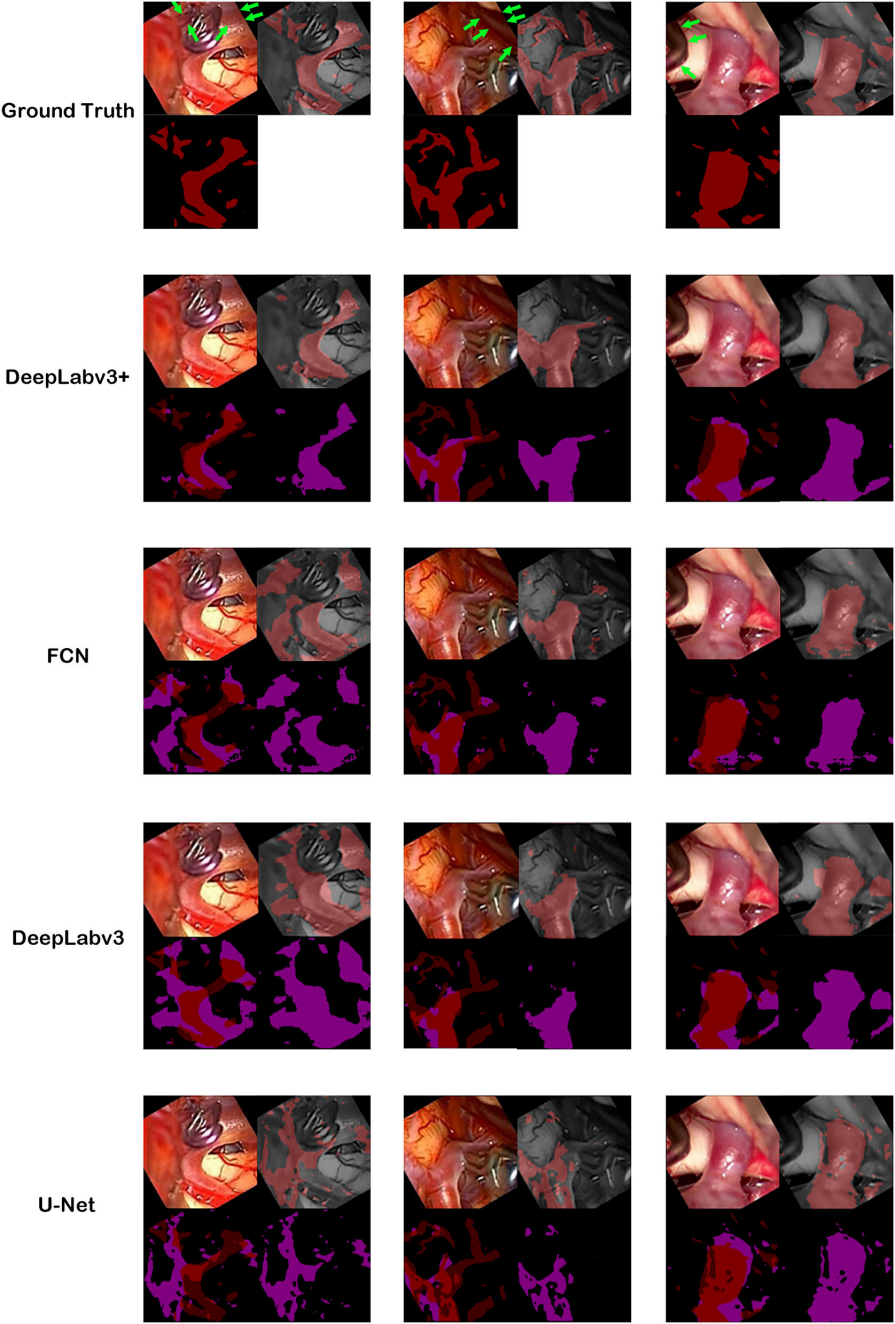
Ground truth and inference results by models. For the first row, cerebral artery ground truth was shown and veins which are not included for the ground truth were marked by green arrows. For each box of four 2 × 2 images, the left upper image is the visible light color image from neurosurgical operating microscope video for analyses. The right lower image is the generated map by the model. In the right upper image, the ground truth in the first row boxes or result map from the second row boxes to the last row boxes is overlapped on monotone source image. In the left lower image, ground truth was shown in the first row. From the second row, result map and ground truth were superimposed to show true positive, false positive, false negative and true negative pixels.

The training and evaluation of each model of the four architectures were conducted on the same test dataset of 99 patients with 92 arterial and 7 delayed phases (Figure 3). The test dataset was gathered from 11 patients and randomly selected from 99 patients. A common test-time augmentation was employed to reduce the prediction error. The pixel accuracy (Csurka et al., 2013), mean IoU (Csurka et al., 2013; Rezatofighi et al., 2019; Eelbode et al., 2020), and mean Dice (Eelbode et al., 2020) between the predicted segmentation maps and the ground truth map generated by our method were all calculated. Both the mean IoU and mean Dice metrics were calculated on the binary classes.

## Results

### Best Results and Confusion Matrices

The best model, DeepLabv3+, yielded a mean Dice of 0.795 for the blood vessel segmentation in the 92-patient artery phase group with 84 patients used for training and 8 patients used for validation (Table 2). The mean Dice decreased to 0.7762 among all 99 patients, partly including delayed-phase patients. Confusion matrices, including true positives, false positives, true negatives, and false negatives, are also presented in Table 2. This information is also shown in Figure 3. In segmentation results, arteries could be distinguished from veins (Figure 3).

**Table 2:** Mean accuracies and confusion matrix for the best results.

	**Mean dice**	**Mean IoU**	**Pixel accuracy**	**True positive**	**False positive**	**True negative**	**False negative**
92-artery patient group: 84 artery phase training/8 artery phase validation	0.795	0.660	0.746	10.7%	4.3%	76.8%	8.23%
99-patient group: 88 artery phase + 4 delayed phase train/8 artery phase + 3 delayed phase validation	0.775	0.632	0.763	9.99%	4.67%	76.1%	9.23%

*The DeepLabv3+ algorithm was used. Values in the confusion matrix are the percentages of pixels*.

### Results Based on the Model

The blood vessel segmentation of various network architectures is summarized in Table 3. The U-Net case exhibited the lowest performance for the overall metrics. The other algorithms were based on the ResNet-101 backbone.

**Table 3:** Result comparisons among algorithms.

**Network**	**Mean dice**	**Mean IoU**	**Pixel accuracy**
DeepLabv3+	0.77618	0.63423	0.86286
DeepLabv3	0.74065	0.58813	0.83537
FCN	0.74273	0.59075	0.84306
U-Net	0.67751	0.51231	0.79522

*Resnet-101 backbones were used for three algorithms except U-Net. Training was conducted in the total 99 patient group: 88 patients train group and 11 patients validation group*.

### Results Based on Dataset Size

**Figure 4:**
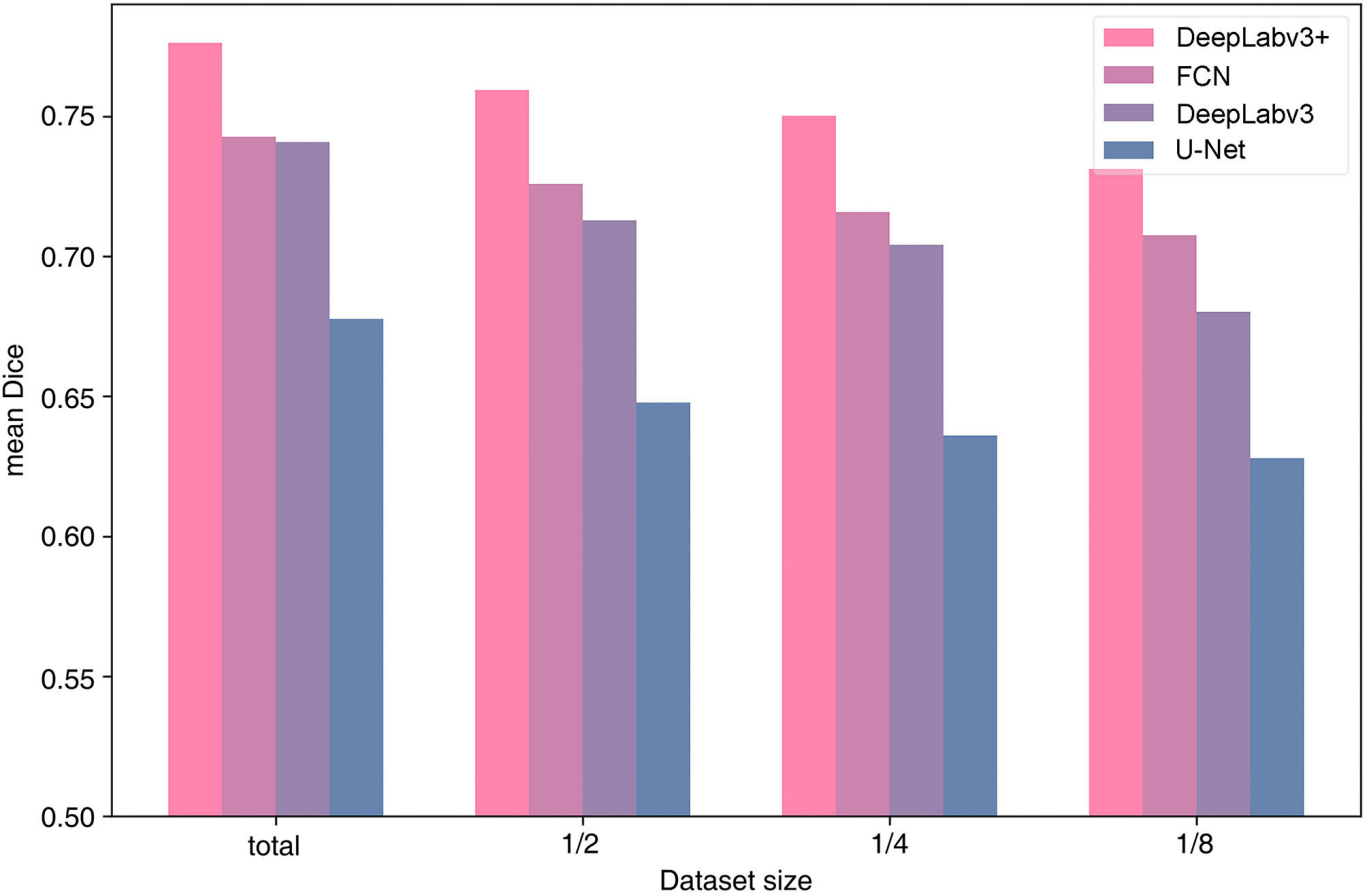
Performance by algorithm and training data size.

We examined the efficiency of the architecture for the dataset size of the 99-patient group. As the dataset increases in size, the tendency to perform better applies to all four architectures. All architectures were tested using four types of dataset sizes (Figure 4). In all cases, DeepLabv3+ resulted in the highest mean Dice value. In particular, more recent architectures, DeepLabv3+ and DeepLabv3, appear to be more accurate.

### Data Augmentation Effects

When no augmentation was used, the accuracy was almost the same, with a difference in Dice score of <0.01. Thus, the decrease in accuracy was extremely small when the Deeplabv3+ algorithm was used without data augmentation.

### Training Hyperparameters

Among the tested hyperparameters in the 99-patient group, a higher learning rate than the default value 10^−10^ and a higher momentum showed slightly better results. For example, when the learning rate was 5 × 10^−10^, the mean IoU was 0.6298. When the learning rate was 10^−8^, the mean IoU was 0.6337. When converted into the Dice score (Park et al., 2019), it was 0.7758. However, when a learning rate is even higher, training was not converged effectively and main IoU accuracy was very low, which is about 0.4–0.5. Thus, learning rate could not be increased further and the currently used learning rate is almost the maximum for the total 99-patient group (Table 4). When the moment is 0.9, the mean IoU is 0.6208. When a momentum of 0.95 was used with the default setting, the mean IoU was 0.6236. The differences were only slight. However, a higher momentum resulted in slightly better results. Thus, as the analysis setting in this study, we finally adopted a higher learning rate and a higher momentum. Both in the 99-patient group and in the 92-patient group, a learning rate higher than 10^−6^ could not be used (Tables 4, 5).

**Table 4:** Hyperparameter settings and results among the 99-patient group with 84 artery phases and 4 delayed phases in the training group.

**Learning**	**Momentum**	**Weight**	**Mean**	**Mean**
**rate**		**decay**	**dice**	**IoU**
10^−6^	0.99	5 × 10^−5^	Low accuracy 0.4–0.5	
10^−7^	0.99	5 × 10^−5^	0.753	0.604
10^−8^	0.99	5 × 10^−5^	0.776	0.634
10^−9^	0.99	5 × 10^−5^	0.771	0.628
10^−10^	0.99	5 × 10^−4^	0.775	0.632
10^−10^	0.95	5 × 10^−4^	0.768	0.624
10^−10^	0.90	5 × 10^−4^	0.766	0.621
10^−10^	0.99	5 × 10^−5^	0.773	0.630

*Test results in 11 patient group of 8 artery phases and 3 delayed phases are shown. Accuracies were about 2.5% lower than homogeneous artery phase 92 patient dataset shown in Tables 2, 5*.

**Table 5:** Hyperparameter settings and results in the 92-patient group with all artery phases.

**Learning**	**Momentum**	**Weight**	**Mean**	**Mean**
**rate**		**decay**	**IoU**	**dice**
10^−5^	0.95	5 × 10^−4^	Low accuracy 0.4–0.5	
10^−6^	0.99	5 × 10^−4^		
10^−6^	0.99	5 × 10^−5^		
10^−6^	0.95	5 × 10^−4^		
7 × 10^−7^	0.99	5 × 10^−5^		
5 × 10^−7^	0.99	5 × 10^−4^		
10^−7^	0.99	5 × 10^−4^	0.637	0.778
10^−8^	0.99	5 × 10^−4^	0.650	0.788
10^−9^	0.99	5 × 10^−5^	0.656	0.792
7 × 10^−10^	0.95	5 × 10^−3^	0.660	0.795
5 × 10^−10^	0.99	5 × 10^−5^	0.660	0.795
5 × 10^−10^	0.90	5 × 10^−5^	0.654	0.791
7 × 10^−10^	0.95	5 × 10^−3^	0.660	0.795
10^−10^	0.99	5 × 10^−4^	0.656	0.792

*Accuracies are for the test results for eight patients with artery phases*.

## Discussion And Conclusion

### Comparison Between Manual and ICG Videoangiography-Based Automatic Ground Truth Characteristics

Automatic ground truth generation based on ICG videoangiography is potentially much more accurate than manual ground truth and, potentially, a gold standard because ICG videoangiography shows the vessels directly (Norat et al., 2019). Manual ground truth is often simplified for conspicuous vessels, and some vessels may be missed during the manual creation of the ground truth (Bano et al., 2021). However, these missed vessels were observed through ICG videoangiography (Figures 1, 3). Thus, we speculate that the automatic ground truth will be more accurate.

However, an automatic ICG-based ground truth may show numerous small vessels, which may be difficult to identify with deep learning from visible light image data (Figures 1, 3). Thus, the ICG-based analysis score cannot be directly compared to a manual ground truth analysis, and the score may actually be lower owing to a higher accuracy of ground truth with much more details about small vessels, which are hard to segmentize based on visible light images (Figures 1, 3).

### Comparisons of Number of Ground Truth Data With the Literature

In previous studies, a few hundred or thousands of manual ground-truth images were used. In a neurosurgical infrared ICG videoangiography grayscale blood vessel segmentation study, 150 training images were used (Jiang et al., 2021). In this previous study, color surgical field vessels were not segmented and only grayscale infrared ICG vessels were analyzed (Jiang et al., 2021). In a recent fetoscopic blood vessel segmentation study, ~345–2,060 video image frames were manually marked (Sadda et al., 2019; Bano et al., 2021). The creation of more ground truth data is limited by the budget, labor, and time. The accuracy is also limited through a manual method. Our automated generation method can increase the number of ground truth data to over 30,000 with limited cost and labor, which is much higher than the number used in the literature.

### Comparison of Segmentation Accuracy With the Literature and Validation Subgroups

During the literature review conducted for comparison, we found that methodological and data variations that can influence the accuracy of the measurements were high and the direct comparisons of the accuracy were not possible. In some previous studies, validation subgroups were not exactly defined and were, potentially, completely or partially conducted for different images from the same patients (Laves et al., 2019; Bano et al., 2021; Jiang et al., 2021). Because anatomical variations within a patient are limited, the accuracy of the algorithm may be overoptimized for a patient and overestimated when evaluated partially with images from the same patient, resulting in a high accuracy considering the number of training images. When testing is strictly applied in a separate group of patients, the accuracy may be lower. In this study, validation was conducted in the patient group, which was completely different from the training group.

### Accuracy of Comparisons Between Neurosurgical Instrument Segmentation and Non-Neurosurgical Surgical Video Anatomy Segmentations

For the neurosurgical field, instrument segmentation was conducted in a previous study (Kalavakonda, 2019). The neurosurgical instrument showed a lower segmentation accuracy (Dice score of 0.769) than the robotic instrument (Dice score of 0.887) (Kalavakonda, 2019).

In previous non-vessel anatomical structure studies, the semantic segmentation accuracy of the uterus, ovaries, and surgical tools with Mask-R-CNN was 0.845, 0.296, and 0.545 (pixel-based mean IoU), respectively (Madad Zadeh et al., 2020). In the fetoscopic study, the overall mean IoUs of anatomical structures, including blood vessels, were 0.58–0.69 (Bano et al., 2021). Anatomical feature segmentation seems quite difficult when considering the accuracies in the literature using the manual ground truth. Thus, the automatic ground truth generation method is promising.

### Comparison of the Segmentation Accuracy With the Literature: Artery Segmentation vs. Vessel Segmentation Including Both Arteries and Veins

In the literature, we found a neurosurgical blood vessel segmentation study (Jiang et al., 2021). However, there are some major differences. The segmentation target was grayscale infrared ICG videoangiography cortical vessels, including both arteries and veins, or anastomosed vessels (Jiang et al., 2021). By contrast, in this study, we only conducted “artery” segmentations in visible light color videos (Figure 1C). Thus, our analysis task was more difficult because of the arteries and the veins that are morphologically similar and need to be distinguished in non-angiographic ordinary color surgical videos with less distinct vessel margins (Figure 1). In the recent neurosurgical cortical vessel segmentation, including arteries and veins, using a limit of 150 training images, the Dice score was 0.80 (Jiang et al., 2021). The main focus of the study was blood flow quantization and not vessel segmentation. Thus, the details of vessel segmentation ground truth and validation methods are unknown. We speculate that validation was conducted partly in the same patient group, and a direct comparison would not be possible.

### Comparison of Segmentation Accuracy With the Literature and the Factors to Consider: Box and Pixel-Based Mean IoUs

In a recent study on surgical instruments and anatomical feature detection studies, the bounding box-based mean IoU (Rezatofighi et al., 2019), including both instruments and anatomical structures, was only 0.56 (Bamba et al., 2021). Because the measurement was the bounding-box-based mean IoU (Rezatofighi et al., 2019), and instruments (Kalavakonda, 2019) are easier to segment than anatomical structures, including blood vessels, the actual pixel-wise semantic segmentation mean IoU of the blood vessels would be even lower than 0.56. This study showed that surgical video anatomical feature segmentation is highly difficult.

### Methodology for Effective Training of Anatomical Structure Semantic Segmentation

During dataset construction and deep learning training, we found that, for deep learning applied to semantic segmentation training for the surgical field, a high-quality dataset is required for the effectiveness of the training.

In many instances, the identities of the surgical anatomy and the anatomical structure are inconspicuous in images because of various factors, including low illumination, poor focus, and a blood-filled or obstructed surgical field (Kalavakonda, 2019; Bamba et al., 2021; Bano et al., 2021). Because low-quality images occur much more frequently than high-quality images in a surgical recording, it is critically important to discard low-quality data.

In addition, morphological characteristics of anatomical structures are variable and need to be classified and allocated to adequately indexed categories. For example, “blood vessels” cannot be trained as a single category, and we focused on large to medium-sized, non-atherosclerotic arteries. Thus, other types of vessels, such as capillaries, cortical vessels (Jiang et al., 2021), and veins, need to be trained in different categories and are beyond the scope of this study.

### Videoangiography Phase and Ground Truth According to Vessel Types

This study shows that, by using visualized vessel-type differences according to the ICG videoangiography phases, vessel types (i.e., from arteries to veins) can be distinguished (Figures 1, 3).

We compared a total of 92 patient groups with all arterial phases (Tables 2, 5) and a total of 99 patients with mostly arterial phases and partly mixed delayed phases. By removing the delayed phase data, the accuracy moderately improved by ~2.5%. This finding shows that ICG videoangiography phase matching is also an important factor for achieving a more accurate analysis. When the delayed phase is mixed with the arterial phase, and the resulting ground truth becomes inconsistent. In this case, most of the ground truth does not contain veins, while some contain veins, resulting in an inconsistency in the ground truth. Thus, the training and test accuracies partly decreased.

### Videos Resolution and Algorithm Training

Because the resolution of each video differs based on whether it is SD and HD, our trained algorithm may be more applicable to various image qualities. These variabilities were expected to enhance the robustness of the model in semantic segmentation in the neurosurgical field. Because a cropped area is used for training, the training data resolution is limited, thereby, also potentially limiting the accuracy. An operating microscope with a higher resolution video recording is required for better accuracy.

### Optimal Architecture for Improvements in Blood Vessel Segmentation and Potential Neural Network Architecture

In several previous studies, U-Net or U-Net variation architectures were used (Kamrul Hasan and Linte, 2019; Bano et al., 2020; Yamato et al., 2020). However, we believe that the accuracy of U-Net is not satisfactory when comparing the results in the literature with our results (Figures 3, 4). In this study, multiple algorithms were compared, as DeepLabv3 and DeepLabv3+ were found to be superior to the conventional U-net (Chen et al., 2018). In DeepLabv3+, a U-net-like encoder–decoder architecture was adopted, and the use of both atrous convolution and a U-Net like architecture is probably beneficial for achieving a better accuracy (Figure 4) (Chen et al., 2018).

In particular, blood vessels typically have an elongated morphology. Thus, we believe that convolutions focused on short distances with small kernels are less effective. DeepLabv3 and DeepLabv3+ use atrous convolutions, comprising longer distanced convolution kernels that may be adequate for elongated morphology objects, such as arms, legs, and blood vessels.

The network backbones used were ResNet-101 for DeepLabv3 and DeepLabv3+ in this study. Using residuals, a type of skip connection used in ResNet would also be important for achieving a better accuracy of blood vessel segmentations, similar to various types of vessel segmentations (He et al., 2016a). In a recent fetoscopic study, the algorithm using a skip connection, ResNet-50, was used and showed relatively fair results (Bano et al., 2021).

The currently used algorithm is probably not the best, and the other types of algorithms, such as the ResNet-152 backbone (He et al., 2016b), Xception backbone (Chollet, 2017), or other architectures may achieve better results. However, the improvement is expected to be small when considering the differences in the accuracy found in the literature (He et al., 2016b; Chollet, 2017).

To find an even more accurate and efficient deep learning algorithm, an automatic machine learning optimization algorithm can be considered (He et al., 2018).

### Data Augmentation Effects

In this study, almost no data augmentation effects were observed. We think this phenomenon can be found when data variability is sufficient in a large dataset, and the algorithm is not prone to over-optimization. For example, no noticeable “jittering” or mirroring augmentation effects were found when FCN segmental segmentation was used for the VOC2012 dataset (Shelhamer et al., 2017).

### Optimal Hyperparameter Setting

The finding in which a higher learning rate and a higher momentum showed that slightly better results are consistent with the literature on hyperparameter tuning (Smith, 2018). However, hyperparameters could not be adjusted further, owing to a failure of the training or divergence. We speculate that the currently used hyperparameter settings are close to optimal.

As discussed in the literature, the hyperparameter setting and the dataset amount and characteristics are inter-related (Smith, 2018), and a higher learning rate can be used in a larger dataset of 99 patients (Table 4) and cannot be used in the 92-patient dataset (Table 5).

### Alternative Methods Used to Assist Vessel Segmentation in the Literature

Alternative methods for assisting in blood vessel detection described in the literature included a change in detection using artery pulsations (Akbari et al., 2009) and Doppler ultrasonography-based imaging of vessels (Kempski et al., 2019). However, artery pulsations and photoacoustic detection margins may not be as precise or as straightforward as the ICG videoangiography margins (Figure 2) and, thus, less adequate for an exactly matched vessel ground-truth generation.

### Potential Further Improvements and Related Studies

Although Otsu's algorithm was used in ICG for threshold selection, deep learning-based threshold selection has recently been reported, such as DeepOtsu (He and Schomaker, 2019). A more accurate threshold selection can be achieved using this method. Co-registration and timeframe matching algorithms can also be improved using deep learning.

From a different perspective, we suggest that the structural complexity of the blood vessels can be interpreted as a graphical theory. The graph neural network (GNN)-based approach for graphical connectivity of the blood vessels may enhance the performance of blood vessel segmentation in the surgical field (Shin et al., 2019).

When considering the inference concepts, for example, Bayesian inference for algorithm development or optimization may also be useful (Doke et al., 2020).

### Importance of Artery Separation From Veins

For clinical and surgical purposes, the separation of arteries and veins is important. Arteries are the usual surgical targets (Norat et al., 2019) and are, thus, more important than veins. If an artery is occluded, it can cause stroke. However, when the vein is occluded, nothing may occur because of the more abundant collateral flows. When only the arteries are segmented, they can be accurately matched to MR or CT arteriography. Separated artery or vein images are easier to discern for diagnostic and interventional purposes, and vein signature contamination decreases the image clarity. Thus, the clinical and diagnostic value of arteries distinguished from veins is high. We first show the separate detection of arteries from veins in a neurosurgical operation image.

### Potential Applications

In the surgical field, blood vessel segmentation can be an important basis for deep learning applications in the surgical field. For example, blood vessels can be used to anchor the registration of the vessel itself or non-vessel structures. Using vessel registration, blood flow quantization (Jiang et al., 2021), and image mosaicking using vessels (Bano et al., 2020) were possible. Marker-based neural navigation systems have weaknesses, such as brain shifting. To address this issue, anatomy-based navigation systems are required. An application to estimate brain shifts during surgery is also possible (Ding et al., 2011). To develop this approach, computer vision blood vessel localization-based navigation of the surgical field can be considered. The detection of blood vessels can be used to design handheld devices aiming to avoid blood vessels for greater safety (Prudente et al., 2017). Our method can be used to analyze the surgical field more accurately. In a recent study on robotic automation, for a surgical assistance device, automated suction and blood flow analysis were applied (Richter et al., 2021). Blood vessel recognition is also potentially helpful in finding the origin of bleeding and applying suction around it or controlling the bleeding focus (Richter et al., 2021). In the long term, image-guided autonomous surgery and surgical assistance systems have potential applications. In the future, surgical assistance and autonomous surgery neurorobotics that can manipulate cerebral vessels will require computer vision to identify the blood vessels.

## Data Availability Statement

The datasets presented in this article are not readily available because of privacy reasons. Data sharing is possible with permission when considering the personal information protection act of Korea and the requests of the operating surgeons. Requests to access the datasets should be directed to the corresponding author.

## Ethics Statement

The study protocol was approved by the Seoul Asan Medical Center Institutional Review Board (IRB No. S2018-0288-0003) and the Ganeung Asan Hospital Institutional Review Board (IRB No. 2018-05-005). Considering the biosecurity and standard safety procedures, this study is a completely retrospective analysis of stored digital data that do not involve human interventions for data collection or the use of biomaterials. Thus, the related risk is minimal, and the study was approved by the IRB Committee when considering the safety concerns.

## Author Contributions

M-sK collected and pre-processed the data with MATLAB, analyzed the dataset and deep learning algorithms, conducted experiments using PyTorch algorithms, and contributed to the writing of the manuscript. JC collected and pre-processed the data with MATLAB, and conducted early experiments using the deep learning algorithms. LH and SL collected and pre-processed the data using MATLAB and partly involved in the deep learning analysis. JA and WP conducted the surgery and IR800 ICG videoangiography and provided operation videos. S-CP led the development of the initial study concepts, designed the research plan, made grant applications and research fund acquisitions, developed a videoangiography-based data acquisition method, collected the data, pre-processed the data with MATLAB, conducted experiments using the Caffe and Tensorflow algorithms on U-Net and PyTorch-based DeepLab V3 and V3+, and contributed to the writing of the manuscript. All authors contributed to the article and approved the submitted version.

## Funding

This work was supported by the National Research Foundation of Korea (NRF) grant funded by the Korean Government (MSIT) (No. NRF-2019R1F1A1063692) (90%), the Medical Research Promotion Program through the Gangneung Asan Hospital funded by the Asan Foundation (2018-B09) (5%), and grant supported by the SNUH Research Fund (No. 04-2019-0770) (5%).

## Conflict of Interest

M-sK, LH, and S-CP were employed by company Deepnoid Inc. Korean Domestic Patent, 10-2195850-0000 by S-CP, JC, SL, and JA is acquired. Partly related international patent application is currently in progress. The remaining author declares that the research was conducted in the absence of any commercial or financial relationships that could be construed as a potential conflict of interest.

## Publisher's Note

All claims expressed in this article are solely those of the authors and do not necessarily represent those of their affiliated organizations, or those of the publisher, the editors and the reviewers. Any product that may be evaluated in this article, or claim that may be made by its manufacturer, is not guaranteed or endorsed by the publisher.
