# Supplementary material for: Deep-Learning-Based Cerebral Artery Semantic Segmentation in Neurosurgical Operating Microscope Vision Using Indocyanine Green Fluorescence Videoangiography
Source: Front Neurorobot. 2022 Jan 12;15:735177. doi: 10.3389/fnbot.2021.735177 (PMC8790180; doi:10.3389/fnbot.2021.735177)
Supplement: Supplementary file 1 [file Table_1.docx]

# Supplementary

*Data Characteristics*

We manually described the dataset into 10 categories, as described in Supplementary Table 1. An image can have multiple characteristics, and nine characteristics are defined. The most frequent characteristic was overall high image quality (25%).

Supplementary Table 1| Training Data Characteristics

| Type | Description | Percentage |
| --- | --- | --- |
| High image quality | Nothing bad about the  images | 25 |
| Duplicate images from the same video | Duplicate data which extracted from the same video exist | 17 |
| Underexposure | The pictures are generally  dark | 14 |
| Anatomical structures which were inconspicuous | The object appears indefinitely | 11 |
| Coregistration difficulty due to inconspicuous margins | The object’s surroundings are complex and seem difficult to match due to inconspicuous margins | 9 |
| Anatomical structures which are sparse too sparse | The object occupies very small portion in the images | 9 |
| Mainly focused vein | Veins are mainly focused and arteries are not conspicuous | 6 |
| Overexposure | Overexposure spot in images | 1 |
| Fragmented | Objects appears in various parts of the images sparsely | 1 |
